# Framing the Issue

**DOI:** 10.1093/geroni/igaa057.2571

**Published:** 2020-12-16

**Authors:** Brian Lindberg

**Affiliations:** GSA, Washington, District of Columbia, United States

## Abstract

The GSA Public Policy Advisor will facilitate a discussion about the 2020 reauthorization of the Older Americans Act with key stakeholders from Washington, DC. Also, the presentation will include perspective on GSA's active role in policy development and the legislative process.

